# Contribution of *Helicobacter pylori* to the Inflammatory Complications of Common Variable Immunodeficiency

**DOI:** 10.3389/fimmu.2022.834137

**Published:** 2022-05-31

**Authors:** Adriana Motta-Raymundo, Pedro Rosmaninho, Diana F. Santos, Ruben D. Ferreira, Sara P. Silva, Cristina Ferreira, Ana E. Sousa, Susana L. Silva

**Affiliations:** ^1^ Instituto de Medicina Molecular João Lobo Antunes, Faculdade de Medicina, Universidade de Lisboa, Lisboa, Portugal; ^2^ Centro de Imunodeficiências Primárias, Centro Académico de Medicina de Lisboa, Lisboa, Portugal; ^3^ Hospital de Santa Maria, Centro Hospitalar Universitário Lisboa Norte, Lisboa, Portugal

**Keywords:** *Helicobacter pylori*, common variable immunodeficiency (CVID), gastric cancer, inflammatory CVID complications, inborn error of immunity (IEI), primary immunodeficiency

## Abstract

Common Variable Immunodeficiency (CVID), the most prevalent symptomatic primary immunodeficiency, is frequently associated with severe inflammatory complications that determine its morbidity and mortality. We hypothesize that *Helicobacter pylori* (HP), a very common worldwide infection, may contribute to the clinical and immune phenotype of CVID. We stratified 41 CVID patients into HP+ (n=26) and HPneg (n=15) groups, according to previous urease breath test and/or gastric biopsies, and compared their clinical manifestations and immune profile evaluated by flow cytometry. No genetic variants with known potential impact in HP infection were found upon WES/WGS. Gastric complications were significantly more frequent in HP+ patients. Importantly, the six CVID patients with gastric cancer were infected with HP. In contrast, a significantly higher frequency of cytopenias was observed in the HPneg. Moreover, HP+ did not feature higher prevalence of organ auto-immunity, as well as of lung, liver or intestinal inflammatory manifestations. We observed the same B-cell profiles in HP+ and HPneg groups, accompanied by marked CD4 and CD8 T-cell activation, increased IFNγ production, and contraction of naïve compartments. Notably, HP+ patients featured low CD25 despite preserved Foxp3 levels in CD4 T cells. Overall, HP impact in CVID inflammatory complications was mainly restricted to the gastric mucosa, contributing to increased incidence of early onset gastric cancer. Thus, early HP screening and eradication should be performed in all CVID patients irrespective of symptoms.

## Introduction

Common variable immunodeficiency (CVID), the most frequent symptomatic primary immunodeficiency (PID), is frequently diagnosed in adulthood, with a monogenic basis being identified in a minority of the cases (1). CVID patients may only present recurrent infections, but up to 50% of them also feature inflammatory complications (2), such as lymphoproliferation, granulomatous lymphocytic interstitial lung disease (GLILD), liver regenerative nodular hyperplasia (LRNH), enteropathy, autoimmune cytopenia, other autoimmune diseases, and increased risk of malignancy, particularly gastric cancer and lymphoma (2). Patients with inflammatory complications feature poorer prognosis (2). Notably, gastric cancer was shown to occur 15 years earlier than in patients without CVID (3), and to be a main contributor to CVID mortality (4). Moreover, the immunological phenotype is also very heterogeneous, presenting a wide spectrum of inflammatory alterations (2), and T-cell impairments (1), in addition to B-cell imbalances (5), which poses a challenge to distinguish it from combined immunodeficiency (CID) (6). Several persistent infections have been considered as potential contributors to the CVID inflammatory complications, such as Cytomegalovirus (CMV) (6), Epstein-Barr virus (EBV) (5), Human Herpesvirus (HHV) (5) and Norovirus (7).

*Helicobacter pylori* (HP) is one of the most common infections worldwide, reported to affect 59% of individuals (8). HP infection is usually acquired in childhood and persists throughout life, leading to microscopic inflammation (9), being symptomatic in only 15% of cases (10, 11). Nevertheless, HP infection is directly related to the development of gastroduodenal diseases, namely gastritis, autoimmune atrophic gastritis, intestinal metaplasia, and gastric cancer (4). The World Health Organization has classified HP as a group 1 carcinogen leading to gastric adenocarcinoma (12). Although the stomach is considered an extremely hostile environment for microorganisms, HP uses a wide range of mechanisms to facilitate the colonization and infection of gastric mucosa (13). Local inflammation triggered by HP plays an important role in both innate and adaptive immunity, mainly mediated by polymorphonuclear cells, and Th1 and Th17 CD4 T cells, respectively (14). Thus, HP triggers the development of exaggerated local inflammation that may impact on gastric epithelial cells function and facilitate gastric carcinogenesis (14). Gastric damage varies with the HP strain, environmental and genetic host factors (13). Genetic polymorphisms of patients infected with HP can directly influence individual variation in the magnitude of the inflammatory gastric response and thus contribute to different clinical outcomes (15).

Several studies have also investigated the putative role of HP in the development of non-gastrointestinal diseases, namely autoimmune diseases such as thyroiditis, psoriasis, neuromyelitis optica and autoimmune cytopenias (16). Immune thrombocytopenic purpura (ITP) is the autoimmune disorder that has been more frequently associated with HP infection (17), and the current clinical guidelines for HP treatment recommend HP screening and eradication in patients with ITP (18).

There are very limited data regarding the prevalence of HP infection in CVID and its impact on the clinical manifestations and immune phenotype (4, 19, 20). The scarce data available suggest an HP prevalence in CVID similar to the general population (20), but studies evaluating systematically the HP status in CVID cohorts, as well as its possible relationship with the genetic background are lacking (4, 19, 20). Given the high impact of gastric cancer on CVID mortality, and the contribution of inflammatory and autoimmune manifestations, including thrombocytopenia, to CVID morbidity, we asked whether HP infection is associated with particular clinical, genetic and immunological profiles of CVID patients.

## Methods

### Study Population

We selected 41 patients with a clinical diagnosis of CVID (1)featuring clinical inflammatory phenotypes and with a genetic evaluation by whole genome sequencing (WGS) or whole exome sequencing (WES). All patients were followed at the adult PID outpatient clinic of Centro Hospitalar Universitário Lisboa Norte (CHULN)/Hospital de Santa Maria by clinicians that are members of the research team and were responsible for collecting clinical information and written informed consents from all participants. Individual clinical, epidemiological and genetic data are depicted in **Tables 1** and **2**. The diagnosis of Granulomatous Lymphocytic Interstitial Lung Disease (GLILD), Liver Regenerative Nodular Hyperplasia (LRNH), and Gastric Disorders, namely Autoimmune Gastritis, Intestinal Metaplasia and Gastric Cancer, were based on organ biopsies. CVID-associated Enteropathy was defined as chronic inflammation in gut histology and/or malabsorption in the absence of pathogen isolation in stools or biopsies. The standard clinical criteria were used for the diagnosis of organ-autoimmunity and cytopenia. All the available clinical data were considered to identify persistent infections, meaning isolation of a microbial agent twice after appropriate treatment. The most frequent infections were encapsulated bacteria, CMV, EBV and Norovirus. HP infection was diagnosed based on urea breath test and/or gastric biopsies, following the methodology of the guidelines for HP diagnosis and treatment (21). All the 41 patients were investigated for HP infection and were divided into two groups, namely HP+, with past/current evidence of HP infection, and HPneg. A control group of 15 healthy subjects (median age 33 years (y) [28y-61y], 10 females) was studied in parallel for the immunological evaluation. The study was approved by the ethical board of CHULN/Faculdade de Medicina da Universidade de Lisboa (FMUL)/Centro Académico Medicina de Lisboa (CAML). All individuals provided written informed consent.

**Table 1:** Clinical and epidemiological data of the HP+ patients.

Patient ID	Sex	Age (y)	Symptom onset (Age)	CVID diagnosis (Age)	HP diagnosis (Age)	Follow-up (y)	Immune profile (Age)	Lung Persistent Bacteria	Gut Persistent Bacteria	Persistent viral Infection	GLILD	LNRH	Cytopenia	Organ autoimmunity	Enteropathy	Gastric cancer	Intestinal metaplasia	Autoimmune gastritis
1	M	53	10	37^*^	43	16	45						Evans		x		X	x
2	F	30	<10	21	22	9	25	x			x			DM1, Thyroiditis	x		X	
3	F	41	<10	16	41	25	37	x		x	x	x	Evans		x		X	
4	M	45	<10	39	35	6	42								x		X	x
5	M	43	37	37	43	6	38		x					Vitiligo			X	
6	M	43	<10	33	33	10	39		x					Vitiligo			X	x
7	F	49	<10	37	39	12	42		x					RA			X	
8	F	51	<10	37	41	14	40	x							x		X	
9	F	41	<10	31	40	10	46	x						Thyroiditis	x		x	
10	M	61	<10	22	58	39	56		x							X		x
11	F	40	16	18	18	22	34		x	x	x	x				X		x
12	M	Died 45	7	22^*^	41	23	40	x			x		Evans		x	X		x
13	M	52	<10	21	43	31	40							Psoriasis	x			
14	F	65	<10	54^*^	55	11	57		x				Evans	RA				
15	F	44	27	27	54	17	53	x					ITP					
16	F	49	29	31	38	18	46							Thyroiditis	x			
17	F	42	4	5	33	37	30		x					Thyroiditis				
18	M	53	<10	40	43	13	41	x	x									
19	F	Died 56	22	42	52	14	49	x	x			x	ITP	Vitiligo				
20	F	Died 79	35	49^*^	73	30	71		x				ITP				x	x
21	M	Died 50	19	19	39	31	50	x	x	x				Psoriasis		X		x
22	M	55	31	39	50	16	52		x			x					x	
23	F	Died 40	13	21	36	19	36	x	x				ITP			X		x
24	M	82	17	54	72	28	72								x		x	
25	M	21	11	15	20	6	20	x		x	x		Evans		x			
26	F	Died 31	<10	20	30	5	25		x	x	x	x	Evans	Pancreatitis, RA		X		x

*Detectable IgA serum levels. y, years; CVID, Common Variable Immunodeficiency Disorders; GLILD, Granulomatous Lymphocytic Interstitial Lung Disease; LHNR, Liver Regenerative Nodular Hyperplasia; ITP, Immune Thrombocytopenia; DM1, Diabetes Mellitus Type 1; RA, Rheumatoid Arthritis like.

### Flow Cytometry

Immune phenotype studies were performed at diagnosis and repeated throughout patient follow-up, by flow cytometry using previously described protocols (22–24). Briefly, whole blood was stained with monoclonal antibodies, red blood cells were lysed using BD FACS Lysing Solution (BD Biosciences), and a minimum of 100,000 lymphocytes per sample was acquired (FACSCalibur, FACSCanto and LSR-Fortessa X-20 flow cytometers, BD Biosciences, San José, CA), with data analysed using CellQuest Software (BD Biosciences) and FlowJo Software (Tree Star Inc., Ashland, OR). In parallel, peripheral blood mononuclear cells (PBMC) were isolated by Ficoll-Hypaque density gradient (Amersham Pharmacia Biotech, Uppsala, Sweden) immediately after blood collection, and used to assess cytokine production upon culture with phorbol myristate acetate (PMA; 50ng/mL, Sigma-Aldrich) plus ionomycin (500ng/mL; Calbiochem, Merck Biosciences, Nottingham, U.K.), in the presence of brefeldin A (10µg/mL; Sigma-Aldrich) for 4 hours, followed by surface staining, fixation, permeabilization, and intracellular staining, as described (24). The representative study of each patient included in the analysis is depicted in **Tables 1**, **2**. The parameters selected were: B-cell subsets defined by the expression of IgD, IgM, CD27, CD38 and CD21; T-cell populations defined by naïve/memory markers (CCR7, CD27, CD45RA, CD45RO), activation markers (HLA-DR and CD38), ability to produce cytokines (IL-2, INFγ and IL-17), as well as regulatory CD4 T-cell markers (FOXP3 and CD25).

**Table 2:** 

Patient ID	Sex	Age (y)	Symptom onset (Age)	CVID diagnosis (Age)	HP diagnosis (Age)	Follow-up (y)	Immune profile (Age)	Lung Persistent Bacteria	Gut Persistent Bacteria	Persistent viral Infection	GLILD	LNRH	Cytopenia	Organ autoimmunity	Enteropathy	Gastric cancer	Intestinal metaplasia	Autoimmne gastritis
27	F	54	35	39	NA	15	52	x						RA	x		x	x
28	F	57	<10	33	NA	24	45					x	ITP	Sjogren, Vitiligo, Thyroiditis	x		x	x
29	M	48	19	39	NA	9	41	x	x			x	ITP	RA				
30	F	51	30	37	NA	14	40	x							x			
31	F	38	<10	14^*^	NA	24	31	x		x	x		ITP					
32	F	52	<10	38	NA	14	40			x				Psoriasis, RA	x			
33	F	31	7	10	NA	21	19	x	x		x		ITP					
34	F	41	13	36	NA	5	38	x		x		x	ITP	Thyroiditis	x			
35	M	27	1	5	NA	22	24						ITP		x			
36	M	47	<10	30	NA	17	35		x			x	ITP					
37	F	41	21	21	NA	20	29	x	x	x	x		ITP					
38	F	38	<10	10	NA	28	29		x									
39	M	42	21	34^*^	NA	8	34	x	x					RA				
40	M	32	6	18	NA	14	20											
41	F	41	<10	20	NA	21	34						AIHA	RA	x			

*detectable IgA serum levels. Y, years; CVID, Common Variable Immunodeficiency Disorders; GLILD, Granulomatous Lymphocytic Interstitial Lung Disease; LHNR, Liver Regenerative Nodular Hyperplasia; IP, Immune Phenotype; ITP , Immune Thrombocytopenia; AIHA, Autoimmune Hemolytic Anemia; DM1, Diabetes Mellitus Type 1; RA , Rheumatoid Arthritis-like.

### Genetic Analysis

WGS/WES were performed in genomic DNA extracted from peripheral blood. WES libraries were constructed using the Agilent Sure Select Human All Exon 50 Mb kit, and sequenced on a Hiseq2000 Illumina sequencer to a 100x sequencing depth (BGI-Shenzhen, China). WGS samples were sequenced to an average read depth of 30x (BGI-Shenzhen). The sequence reads were mapped to the reference GRCh37 genome using the BWA-MEM aligner. SNPs and Indels were called following the GATK4 germline short variant calling pipeline (25), and annotated using the Variant Effect Predictor (26). We analyzed variants in a panel of 16 genes that were previously associated with gastric diseases and HP infection (15), and in a panel of 430 genes that have already been described as monogenic causes for primary immunodeficiency according to current IUIS classification (27). We filtered variants according to consequences, and excluded intron and synonymous variants. Then, we filtered files and selected only variants with high, moderate or modifier impact prediction in respect to protein structure, with an allele population frequency below 1%, and classified them as pathogenic or likely pathogenic at the Varsome platform (28), according to ACMG criteria (29–32).

### Statistical Analysis

Statistical analyses were performed using GraphPad Prism, version 8.0 (GraphPad Software Inc., La Jolla, CA, USA). Two group comparisons were performed using Fisher’s exact test to demonstrate independence from clinical parameters. Ordinary One-way ANOVA and Fisher’s Least Significant Difference (LSD) test was used to analyze the immunological profile and differences between Healthy Cohort, HP+, and HPneg groups. Results are expressed as median with range in brackets, and *p*-values <0.05 were considered significant.

## Results

**Figure 1:**
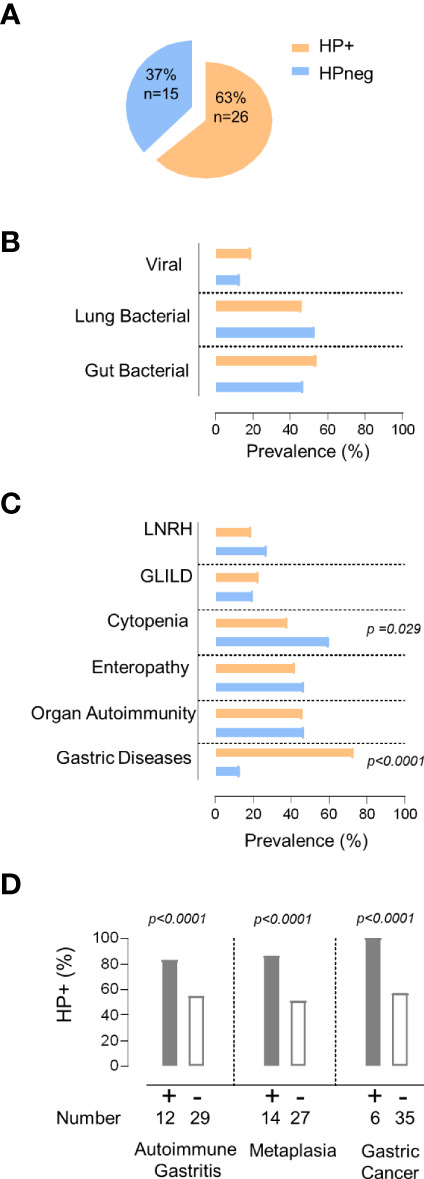
*HP infection and clinical manifestations.* **(A)** Frequency of HP infection in the total cohort. **(B, C)** Comparison between the group of patients with evidence of HP infection (orange) with the group without HP infection (blue) regarding: **(B)** prevalence of persistent viral and bacterial infections, **(C)** non-infectious manifestations. **(D)** Prevalence of HP infection in the group of patients with gastric diseases and those without. The statistical test used was Fisher´s exact test and *p* values <0.05 are shown. LNRH, Liver Nodular Regenerative Hyperplasia; GLILD, Granulomatous Lymphocytic Interstitial Lung Disease.

In order to evaluate the impact of HP on the clinical and immune profile of CVID patients, we first asked whether they feature increased susceptibility to HP infection. The prevalence of HP infection in our cohort (**Figure 1A**) was comparable to the worldwide population (20). However, it is important to emphasize that the HP prevalence is known to be much higher in the Portuguese population (33), reaching levels above 70% in young adults (18y-30y), which further increases with aging (33, 34).

Patients were divided into two groups according to HP status (**Figure 1A**). Remarkably, the two groups did not significantly differ in terms of age (HP+ 46y [21–65y] vs HPneg 43y [27–67y]), sex (54% females in HP+ vs 67% in HPneg), symptom onset before 18y of age (79% of HP+ vs 67% of HPneg), and age at CVID diagnosis (HP+ 30y [5–54y] vs HPneg 26y [5–39y]).

Next, we compared the frequency of other persistent infections that may contribute to the observed clinical and immune disturbances (5–7), and found similar frequencies of patients with persistent bacterial pulmonary and/or gut infections, as well as with chronic viral infections in HP+ and HPneg groups (**Figure 1B**; **Tables 1**, **2**).

Then, we compared the prevalence of the main inflammatory manifestations known to clearly impact on CVID morbidity and mortality, namely GLILD, LRNH, gastric diseases, enteropathy, organ autoimmunity, and cytopenias (**Figure 1C**). Notably, HP+ only featured significantly higher frequency of gastric diseases (HP+ 73% vs HPneg 13%; *p <*0.0001; **Figure 1C**), whereas cytopenias were significantly more frequent in the HPneg group (HP+38% vs HPneg 62%; *p* =0.029; **Figure 1C**).

Considering the higher frequency of gastric diseases in the HP+ group, we further compared the frequency of autoimmune gastritis, intestinal metaplasia, and gastric cancer between HP+ and HPneg, and found that all were significantly increased in HP+ (*p*<0.0001; **Figure 1D**). Importantly, there were no cases of gastric cancer without HP infection (**Figure 1D**), supporting a determinant role of HP infection in the pathogenesis and very early onset of gastric cancer in CVID (4, 19). In this regard, it is worth noticing that the median age at the first HP test was 41 [18-73y], thus with a median 10y [0-36y] delay after CVID diagnosis, which was largely done in other hospitals, before reference to our primary immunodeficiency outpatient clinic.

To assess a possible contribution of genetic factors to these findings, we intentionally included patients with available WGS/WES data, and our search did not reveal pathogenic or likely pathogenic variants in a list of genes previously associated with gastric diseases and HP infection (15).

We also evaluated a panel of 430 genes considered to be related to inborn errors of immunity (IEI) in the most recent IUIS classification (27), and confirmed the absence of mutations with validated functional impact.

**Figure 2:**
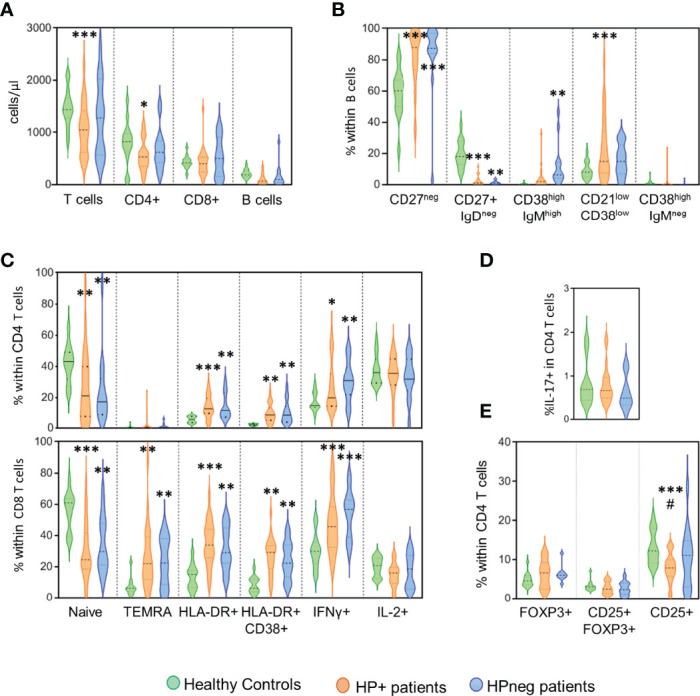
*HP infection and immune phenotype*. Comparison of the groups of patients with evidence of HP infection (orange), without HP infection (blue) and healthy controls (green) regarding the following immunological markers evaluated by flow cytometry: **(A)** Number of circulating T and B cells; **(B)** B-cell subsets; **(C)** CD4 T-cell subsets (top) and CD8 T-cell subsets (bottom); **(D)** frequency of CD4 T cells able to produce IL-17; and **(E)** Frequency of CD4 T cells expressing regulatory T-cell markers. Ordinary One-way ANOVA and Fisher’s Least Significant Difference (LSD) test were used for the statistical analysis and the following *p* values are shown: ***<0.0001; **<0.01; *<0.05 as compared to healthy controls; ^#^<0.05 as compared between patient groups. TEMRA, Terminally effector memory RA.

Finally, we asked whether the presence of HP infection impacted on the immune profile of CVID patients (**Figure 2**). The HP+ and HPneg groups did not significantly differ regarding patient age at the time of immune evaluation (41y [20-72y] in HP+ *vs* 34y [19-52y] in HPneg).

The HP+ group showed significantly reduced numbers of circulating T cells (*p <*0.0001), particularly CD4 T-cell counts (*p <*0.05), when compared to healthy controls (**Figure 2A**). Of note, no significant differences in the CD4/CD8 ratio were observed between the three groups (1.53 [0.29-7.71] in HP+; 1.39 [0.38-6.78] in HPneg; 2.12 [1.30-3.90] in controls). There was a significant loss of switched memory B cells in both HP+ and HPneg in comparison with controls, although the increase in transitional B cells was only observed in HPneg (*p <*0.001) and, conversely, the expansion of the population expressing low levels of CD21 only was only found in HP+ (*p <*0.0001) (**Figure 2B**). These results suggest possible differences in the pathways of B cell impairment according to HP status, despite the lack of statistical differences in these B cell populations when HP+ and HPneg groups were compared (**Figure 2B**).

Regarding T cells, there was a marked contraction of the naive compartment in both HP+ and HPneg in comparison to controls, with significant increases in the expression of activation markers and IFNγ production (**Figure 2C**). The profile of disturbances was very similar in the two patient groups in both CD4 and CD8 T cells, suggesting that HP infection was not a determinant player in the persistent heightened activation of circulating T cells (**Figure 2C**). We didn´t observe significant impairments in IL-17 production in our HP+ cohort (**Figure 2D**), despite its role in HP protection (14), as well as in gut mucosal inflammation (35, 36). Regulatory CD4 T cells, as defined by the expression of FOXP3 in the presence or absence of CD25, were also found to be preserved in both HP+ and HPneg groups (**Figure 2E**). However, there was a significant decrease in the frequency of cells expressing CD25 within total CD4 T cells in HP+ compared to both the control (*p <*0.0001), and HPneg (*p <*0.05) groups (**Figure 2E**). Therefore, HP infection in CVID patients is associated to low CD25 in CD4 T cells, a feature that has been linked with more severe disease in CVID (37, 38).

## Discussion

We evaluated the contribution of HP infection to the inflammatory manifestations of CVID patients and found that HP has a severe impact in all gastric diseases and is linked to early onset gastric cancer. However, no association was found with higher prevalence of regenerative nodular hyperplasia of the liver, lymphocytic infiltrations in the lung or gut, organ specific autoimmunity or cytopenias. Regarding the impact on the immunological profile, we observed that a past history of HP infection was associated with expansion of B cells expressing low levels of CD21, as well as CD4 T cells with reduced CD25 levels, both surrogate markers of severe CVID (37–42).

Interestingly, our data support that CVID patients are not more susceptible to HP infection. In fact, HP is highly prevalent in Portugal (33), achieving frequency levels much higher than the prevalence we observed in our CVID cohort. Our results are in line with previous reports of no increase in HP prevalence, both in CVID and in IgA deficiency (43, 44).

The CVID hallmark is impaired immunoglobulin production, with most of the individuals featuring no IgA, which is a main player in mucosal protection (1). These defects in combination with T-cell impairments are thought to explain the high incidence of mucosal infections in CVID. However, secretory IgA deficiency does not seem to play a prominent role in the establishment of HP infection, with *in vitro* studies showing that it has a minimal inhibitory effect on HP adherence to gastric epithelial cells, and suggesting that mucosa invasion and the local inflammatory response depend on the type of HP strain (45) and on the intensity of patients´ immune response (2). In the future it will be very important to gather data on the HP strains present in our CVID cohort, given the major impact of HP in early onset gastric cancer.

In order to be able to evaluate the potential contribution of variants in genes known to be involved in HP infection and gastric diseases, we only included in our study patients with WGS/WES data. We did not identify potential pathogenic variants in genes that have been described as related with HP infection and gastric diseases (15). Additionally, we excluded the presence of mutations with confirmed functional impact in the genes previously associated with IEI (27).

Past studies outside the context of primary immunodeficiency suggest an association of HP infection with psoriasis, autoimmune thyroiditis, neuromyelitis optica, and particularly with immune thrombocytopenic purpura(ITP) (16, 46). The high frequency of ITP in patients with HP infection is thought to be explained by molecular mimicry between HP and platelets (17), with HP treatment being currently recommended by the American Society of Hematology and by clinical guidelines for the treatment of HP infection in patients with immune thrombocytopenic purpura (18). Cytopenias, particularly thrombocytopenia, are highly prevalent in our cohort, as well as other organ-autoimmune manifestations, in agreement with the data from other cohorts with severe CVID (47). Contrary to our expectation, the HP+ group did not feature a higher prevalence of autoimmune diseases, and we observed an apparent opposite effect, with significantly higher number of patients with cytopenia in the HPneg group, suggesting distinct mechanisms underlying the thrombocytopenia in CVID. Nevertheless, this finding should be cautiously valued given the limitations in diagnosing autoimmune cytopenia in patients with antibody deficiency, and the difficulty in excluding the contribution of hypersplenism due to lymphoproliferation and/or portal hypertension, frequently associated to liver nodular regenerative hyperplasia in CVID.

In parallel with the lack of impact of HP status in the prevalence of inflammatory/autoimmune manifestations beyond gastric diseases, we found significant B-cell disturbances, as well as contraction of the naïve T-cell compartment and marked T-cell activation and IFNγ production in both groups, in agreement with their severe clinical profiles (2). It is plausible that the size of our sample and the wide individual variation of the immunological parameters limited the ability to reveal a possible impact of the HP infection in the circulating lymphocytes of these patients.

Notwithstanding this lack of significant differences between HP+ and HPneg groups, a significant expansion of the population of B cells with low levels of CD21, and reduced expression of CD25 in CD4 T cells was found only in the HP+ as compared to healthy controls.

Decreased CD25 levels in CD4 T cells, irrespective of being regulatory or conventional, was previously described both in CVID (37, 38) and in Systemic Lupus Erythematous (48), particularly in stages of high disease activity with exacerbated inflammatory processes. There is still poor understanding of the role of the subset of quiescent conventional CD4 T cells expressing CD25 in healthy subjects (49). Nevertheless, its functional relevance is becoming more obvious upon the reports of significant decrease of this population in severe SLE (48) and CVID patients (50) suggesting that the loss of CD25 may impact on immune dysregulation independent of regulatory T cells.

Loss of CD21 expression is a hallmark of tissue-like B cells and is linked to persistent immune activation (41, 42). In CVID, the expansion of CD21^low^ B cells has been significantly associated with splenomegaly and autoimmune diseases (41), although the role of CD21^low^ B cells in the pathogenesis remains unclear. Interestingly, our previous data on CVID patients with gastric cancer (51) did not show B-cell infiltration in the biopsies despite these individuals, which are included in our HP+ group, featured the highest frequencies of circulating CD21^low^ B cells.

The HP associated chronic inflammation of gastric mucosa involves marked local Th1 and Th17 inflammatory responses, which likely contribute to changes in the function of gastric epithelial cells that contribute to the gastric carcinogenesis process (14). To the best of our knowledge, there are no data addressing specifically the HP impact on the gastric mucosa of CVID patients and the putative role of IFNγ/IL-17. We found no impairments in IL-17 production by circulating T cells, but it is necessary to evaluate its production in the gastric mucosa.

It would be also relevant to evaluate whether the impact of HP is mainly secondary to the dysregulated immune response related to the genetic background of this population or if the virulence of HP strain plays a role in the gastric disease. There are currently no data on HP strain prevalence in CVID patients.

HP impacts particularly the gastric mucosa, where it is a known risk factor for the development of gastric cancer (21). Gastric cancer is the fifth most frequently diagnosed malignancy in the world and the third leading cause of malignancy-related death in the general population (51). In CVID, gastric cancer is a leading cause of morbidity and mortality, being 10-fold more frequent and usually diagnosed at least 15 years earlier than in the general population (51).

It is worth emphasizing that all gastric cancer cases in our cohort were associated with HP as well as most of the cases of other gastric disorders, highlighting the need of active screening, treatment, and eradication of HP in CVID patients (3, 18). Importantly, we observed in our cohort that the mean time interval between the diagnosis of CVID and the first exam performed to detect HP infection was 10 years, and that most patients tested positive for HP at the first screening test. Therefore, earlier and more regular screenings for HP are mandatory, starting at the time of CVID diagnosis even in adults and children without dyspeptic symptoms. This screening should be continuously repeated, since early interventions may ensure reduction in morbidity and mortality of these patients.

Past studies that evaluated gastric cancer and/or HP in CVID cohorts did not systematically evaluated HP infection (3, 19). Our study is the first to our knowledge that performed HP screening following the previous recommendations for HP investigation in CVID patients (3), and stratified them according to HP status. Moreover, we selected only patients with inflammatory complications to homogenize the cohorts to address the HP impact in the different type of clinical manifestations, and combined genetic and immunological data to provide a novel comprehensive approach of the HP role in CVID. We demonstrate that, in contrast to other clinical settings, the impact of HP infection is mainly restricted to the gastric mucosa, playing a minor role in other inflammatory complications. We also expose the severe disruption caused by HP in the gastric mucosa of CVID patients, leading to a very early onset of severe gastric inflammatory diseases and gastric cancer, highlighting the need of active screening and eradication of *Helicobacter pylori*, irrespective of dyspeptic symptoms.

## Data Availability Statement

The datasets presented in this article are not readily available because of ethical and privacy restrictions. Requests to access the datasets should be directed to the corresponding author.

## Ethics Statement

The studies involving human participants were reviewed and approved by Ethical Board of CHULN/Faculdade de Medicina da Universidade de Lisboa (FMUL)/Centro Académico Medicina de Lisboa (CAML). The patients/participants provided their written informed consent to participate in this study.

## Author Contributions

AM-R, PR, AES, and SLS designed the study. AM-R, RF, SPS, and SLS collected clinical data. CF reviewed pathological data. DS and AES performed the immunological studies. AM-R and PR performed genetic analyses. AES and SLS supervised the study. AM-R, SLS and AES wrote the paper. All authors contributed to the article and approved the submitted version.

## Funding

This work was funded through the grant PAC - PRECISE - LISBOA-01-0145-FEDER-016394, co-funded by FEDER through POR Lisboa 2020 - Programa Operacional Regional de Lisboa PORTUGAL 2020 and Fundação para a Ciência e a Tecnologia (FCT). AM-R received a PhD scholarship from FCT.

## Conflict of Interest

The authors declare that the research was conducted in the absence of any commercial or financial relationships that could be construed as a potential conflict of interest.

## Publisher’s Note

All claims expressed in this article are solely those of the authors and do not necessarily represent those of their affiliated organizations, or those of the publisher, the editors and the reviewers. Any product that may be evaluated in this article, or claim that may be made by its manufacturer, is not guaranteed or endorsed by the publisher.
